# An Evaluation of MINDFIT—A Student Therapeutic Running Group as a Multi-Layered Intervention in the United Kingdom

**DOI:** 10.3390/nursrep13010042

**Published:** 2023-03-13

**Authors:** Jan Gurung, James Turner, Elizabeth Freeman, Charlotte Coleman, Susan Iacovou, Steve Hemingway

**Affiliations:** 1 Student Wellbeing Service, Sheffield Hallam University, City Campus, Sheffield S1 1WB, UK; 2 Department of Nursing and Midwifery, College of Health and Human Sciences, Sheffield Hallam University, Collegiate Campus, Sheffield S10 2BQ, UK; 3 Department of Psychology, Sociology and Politics, College of Sciences and Arts, Sheffield Hallam University, Heart of the Building, Collegiate Campus, Sheffield S10 2BQ, UK; 4 Department of Life Sciences, Psychology, Brunel University, Kingston Lane, Uxbridge, London UB8 3PH, UK; 5 Department of Health and Human Sciences, University of Huddersfield, Queensgate Campus, Huddersfield HD1 3DH, UK

**Keywords:** student wellbeing, running, mental health, green space, mixed methods

## Abstract

Background: Mental health is an ever-increasing concern for UK Universities and is underreported. Creative and dynamic approaches to tackle student wellbeing are important. In 2018, Sheffield Hallam University (SHU) Student Wellbeing Service initiated a pilot study of a therapeutic running programme ‘MINDFIT’ that combined physical activity, led by a counsellor, alongside a psychoeducation approach to support student mental health. Methods: Mixed methods were used which included the Patient Health Questionnaire-9 (PHQ-9), evaluating low mood and depression, and Generalized Anxiety Disorder Scale-7 (GAD-7), evaluating levels of anxiety. Results: A total of 28 students were triaged onto a weekly programme over three semesters. Overall, 86% of the participants completed the programme. A promising reduction in the scores for PHQ-9 and GAD-7 was found at the end of the programme. Focus groups, with student participants, were held to gather qualitative data for analysis. After thematic analysis, three main themes emerged: “Creating a safe community”, “Making progress” and “Pathways to success”. Conclusions: MINDFIT was an effective and engaging multi-layered therapeutic approach. Recommendations identified the importance and effectiveness of the triage process in recruiting students and sustainability of the programme through the continued engagement of students post programme. More research is required to identify the long-term effects of the MINDFIT approach and how applicable it is to higher education contexts.

## 1. Introduction

This paper reports on a pilot university wellbeing initiative to support student mental health and wellbeing. A therapeutic running group, MINDFIT, was developed and evaluated to explore if running and a structured psychoeducation programme could have positive outcomes for students. MINDFIT is based on the ‘Couch to 5k’ 10-week NHS programme [1]. It is a simple form of continuous aerobic exercise of self-selected intensity, with runs taking place in nature. This was combined with psychological support, led by a counsellor who was a trained run leader, supported by a mental health nurse and psychologists, to create a compassionate environment. It followed the principles of Oswald et al.’s [2] referral process, initial intervention model, and encouragement to help integrate running into the student’s lifestyle.

### 1.1. Context

One of the biggest drivers of change within UK universities (notwithstanding the international context) is student mental health, with an estimated 74,000 students with an existing mental health condition entering HE in 2020. There has been an increase of 450% of applicants sharing mental health conditions over the last decade [3]. COVID-19 has compounded the issue with an increase in depression and anxiety noted [4]. University wellbeing services are under increasing demand and pressure to address the varied mental health and wellbeing needs of students in their care. It is important for services to innovate, have a range of wellbeing offers and find new ways to improve mental health. Physical Activity (PA) may be able to fill this gap where need and ability to access services do not match [5], through a creative and holistic approach [6,7]. Early intervention is important to support students to succeed in their studies, especially as students with mental health conditions tend to have lower rates of continuation, attainment and progression into skilled work or further study [8].

De Girolamo et al., [9] note that most mental health problems develop in the first three decades of life. Risks are impacted by the key stage of brain development from child to adult where the brain goes through a process of maturation and the nervous system develops, creating increased vulnerability to stress in young adults [5]. Despite developmental vulnerability, this period also provides the opportunity to develop prevention strategies.

### 1.2. Physical Activity and Mental Health

Prior to the COVID-19 pandemic, studies suggested there was an association between physical activity (PA) and positive mental health [4,10,11], particularly in helping people manage depression [10]. During the pandemic, medical advisors turned a spotlight on the benefits of PA on mental health and this was promoted in government briefings and popular culture. This has also prompted more studies and a growing evidence base. The UK NICE guidelines for depression recognised this and recommended physical activity as a treatment for mild depression [12] and as an intervention referral route for primary care practitioners [13]. The evidence is growing generally on the benefits of PA and exercise on mental health [14]. Whilst PA was not previously seen as a treatment option in traditional mental health services, there is now a developing acceptance that mental health treatment pathways can use PA either as a main therapeutic tool or as an addition to therapy [15].

Several systematic reviews and meta-analyses show positive results for PA as a treatment for depression across a variety of ages [16,17]. A recent meta-review reported that physical activity leads to consistently positive effects on depressive symptoms in both clinical and non-clinical samples of children and adolescents. No negative side effects were noted [18]. Pascoe et al.’s [19] review on prevention of depression in young people aged 12 to 25 described PA as a MH ‘protective factor’, as an opportunity to build coping strategies, reduce stigma and encourage social community. Aerobic exercise has alleviated mild to moderate depression, showing similar effects to medication or cognitive behavioural therapy [20].

PA is also suggested as a positive intervention for anxiety (which is often comorbid with low mood). Whilst the effects of PA on anxiety have not been as prominent, exercise at self-selected intensity has been shown to elicit positive changes in core mood affect and pleasant feelings among people with diagnosed clinical anxiety [21]. More recently, a meta-analysis of randomised controlled trials of young people up to 25 years showed that as with depression, PA has positive effects on anxiety [22]. PA had ‘significantly superior effects on state anxiety’ when compared to a time and attention-controlled group (ibid).

Alongside MH there are benefits of exercise on physiology. For example, negative effects of increased inflammation in the brain and the positive effects of physical activity in reducing this is an evolving area. Inflammation may affect several neurotransmitters in the brain including serotonin which affects motivation and anxiety, and increased levels of cytokine and kynurenine which may contribute to the development of depression [23]. A six-week programme of moderate intensity exercise in a student population saw a decrease in symptoms of depression and a reduction in pro-inflammatory cytokines [24]. Persistent increased levels of kynurenine fell significantly in an experiment with mice that exercised [25]. It appeared that physical exercise reduces inflammatory markers [26].

### 1.3. Running, Mood and Anxiety

Running is a way of relieving the stress of modern society [27,28], and can elicit positive changes, including increased tranquillity and revitalisation [29]. Research suggests that a run at moderate pace can give a four-hour window of improved mood [30]. Runners often talk about experiencing ‘runners high’ and recent scientific findings are beginning to attribute this with endocannabinoids. These are self-produced chemicals, similar to those found in marijuana [31], which can contribute to an improvement in mood. Oswald et al., [32] conclude that running provides an important and economical therapy for those patients with depression and anxiety disorders, and suggest that it is important to integrate into the lifestyle of patients as a regular activity [33]. MINDFIT followed these principles with its referral process, 10-week initial intervention model and encouragement of integrating sustainable running into the participants’ student lifestyle.

### 1.4. Green Exercise

Some forms of exercise, particularly walking and running and outdoor sports, either partially or fully take place in a green space and can be referred to as green exercise. Green exercise has been shown to reduce stress, provide opportunities for restoration, reduce blood pressure, improve mood and concentration, along with promoting feelings of peace and calm across all age groups [34,35]. Greater improvements are seen in those participants experiencing low wellbeing [36]. Greenspace was felt important so was included in the MINDFIT programme design, using a local park for each session.

### 1.5. Parkrun

MINDFIT culminates with a parkrun celebration event and in doing so continues the green exercise approach, connects participants to the local community and introduces them to a way of maintaining their running beyond the MINDFIT programme. Parkrun takes place weekly, all around the world, and is a free, mass participation, 5 km running event [37]. Quirk et al., [38] analysed 60,000 survey returns of 47 questions, and found that parkrun attracts a broad range of the population from young to old, least active to most active, and most deprived to least deprived.

Often people with mental health problems experience exclusion, yet parkrun promotes inclusivity, which has been reported to be beneficial to people with mental health difficulties [39]. It provides accessibility, opportunities for achievement and social support, in outdoor settings, that can contribute to health-enhancing physical activity outcomes [33,40].

MH interventions are needed which are attractive to young people, which reduce stigma and help them overcome mental barriers to taking part. Informal PA groups in non-clinical settings, such as in nature or urban green space, create an ideal environment to achieve this. Evidence from exercise and mental health, green exercise and parkrun research has informed the design of an intervention that aimed to improve university students’ mental health.

## 2. Materials and Methods

### 2.1. Aim

To evaluate the impact of the MINDFIT programme on the wellbeing of student participants.

### 2.2. Objectives

To identify whether MINDFIT improves low mood, depression and anxiety among university students.

To understand the lived experience and benefits of MINDFIT for university students, staff facilitators and sport activators.

### 2.3. Designing the MINDFIT Programme

Based on available evidence, MINDFIT, a 10-week couch to 5 km structured therapeutic running group combining physical activity in nature with a psychoeducation and counselling approach, was developed. It was facilitated by a team, including a counsellor, a mental health nurse and psychologists, a university sports participation manager and chaplaincy staff. The evidence of the positive effects of physical activity and green exercise on mental health [21,30,37] and the importance of providing inclusivity, accessibility, opportunities for achievement, social support and volunteering opportunities in outdoor settings [40] informed the programme.

Sessions were run weekly on Wednesday afternoons, coinciding with the university’s Wellbeing Wednesday campaign, when lectures do not generally take place and time is allowed for health and wellbeing activities.

**Session schedule**

- Meet and greet;
- Run Leader communicated the plan for the session;
- 5-min warm up walk to the park;
- 5 min dynamic stretching and drills—run by a student sports activator;
- 50 min run/walk;
- 5-min warm down walk;
- 55 min psychoeducation, socialising/drinks and snacks.

Participants were divided into groups according to their running competence and fitness, each with a run leader or student sports activator to support. Each group ran the same circular route and adjusted distance and running duration according to fitness. The group reassembled at the end of the course and walked back to campus together as a warm down and to facilitate conversation between participants with different fitness levels. The NHS couch to 5k programme gradually builds fitness, increasing running time and distance and this approach was used incrementally over 10 weeks.

### 2.4. The Therapeutic Approach

Inclusivity and a non-competitive philosophy are at the heart of the programme, providing a safe environment for healing and connection for participants. The programme is designed to accommodate all ages, abilities and fitness levels and is accomplished by runs taking place in local parks followed by refreshments, some psychoeducation and group support. The psychoeducation content is shown in Table 1.

A four-dimensional existential therapeutic model [41] provided a holistic lens to view student wellbeing and programme design. This model enables a wide view of mental health, not limited to cognitive and emotional processes, and consideration of how students responded to their surroundings and influences. Motivational Interviewing techniques (MI) were used throughout the programme in a practical way to encourage participation, help students master their goals and work with their resistance, perceived obstacles and ambivalence [42].

The MINDFIT team of staff and student sports activators had a variety of specialist skills, including England Athletics leader qualifications, experience in running and mental health training. A trained run leader was present at each run and the counsellor and/or a mental health nurse academic were available after each run for brief problem solving, guidance or signposting. Group discussion was guided and at times led by the student participants to be non-intrusive and welcoming, in order to foster a sense of community. Student sports activators who assisted were given Mental Health First Aid training, England Athletics run leader training, and were able to join the SHU Sports Coaching Academy to help enhance their employability. The programme culminated with a parkrun celebration event at the end of the 10-week programme. Alongside the group, students were offered 8 weeks free prescription for exercise, per year, to access the university gym facilities.

### 2.5. Participants

Criteria for inclusion were as follows:

- Age 18 and over;
- Any gender;
- University registered student or a student on a break in study;
- Have the physical ability to walk and run.

Exclusion criterion were as follows:

- Unable to provide informed consent;
- Students expressing a risk to life by making suicidal plans, to prevent causing harm through the intervention.

### 2.6. Recruitment

Recruitment was via advertisement across all levels of study and all courses through emails, when registering with Student Wellbeing, or through referrals from wellbeing practitioners, counsellors, mental health and inclusive support practitioners (N = 48). Other referrals came from the University Medical Centre (N = 5), Student Support Advisors (N = 3) and self-referral through the Student Wellbeing website (Group 3 only, N = 6). The marketing of the programme was encouraging in tone and promoted the message of a non-competitive group for students who wanted to run but did not feel able to join a university sports club or society. Existing runners and non-runners were welcomed as well as those who needed help to start running again. Students who had completed a programme and wanted to remain involved were encouraged to return.

A group breakdown of the sixty-two students referred to the programme is presented in Table 2. Thirty-three were triaged and twenty-eight joined the programme. Five students were triaged out because of personal or study circumstances and twenty-nine referrals did not attend. Twenty-four (86%) of the attenders (6 in Group 1, 9 in Group 2, and 9 in Group 3) completed MINDFIT by attending the minimum number of 6 sessions and took part in a finishing event. Group 4 data are incomplete due to COVID-19 interruption.

Attendance was monitored weekly and to complete the programme students needed to attend a minimum of six sessions, including the celebration event. Students were encouraged to communicate non-attendance and illness was taken into consideration. Those who dropped out of the programme did so between triage and the first session or within the first two weeks of the programme. A variety of reasons were cited including guilt of taking time out of studying, moving house, being unwell, and finding it logistically difficult to attend. Overall, 24 students (19 female and 5 male), 1 PhD student, 2 master’s students and 21 undergraduates, from 17 diverse courses, completed the programme. The Department of Nursing and Psychology attracted the most participants.

### 2.7. Data Collection

A mixed method approach was taken to evaluate MINDFIT, including statistical and qualitative evaluation.

For the qualitative evaluation, three semi-structured focus groups, with between six and nine students, were conducted by a psychologist and the counsellor run leader. A semi-structured interview (SSI) approach was utilised to capture the meaning in the participants’ own words [43]. SSIs allow a flexible approach to data gathering and provide a starting point for understanding the individual’s experiences and meanings [44]. Questions explored what factors were most important, including joining the group, the physical and emotional response to running, the structure of the programme, post run sessions, communications, and the celebration parkrun. Focus groups were ‘in person’ and audio recorded. To help understand the overall experience of all parties involved in MINDFIT, qualitative surveys were used to gather the views of the student sports activators and staff who had referred students to the group, who subsequently received feedback from students during follow-up wellbeing or counselling sessions.

Qualitatively, inductive thematic, team-based analysis, was utilised [45] for the focus group data. Six stages of inductive thematic analysis were completed to increase trustworthiness of the data [46]. Themes were triangulated by three researchers who analysed, discussed, reviewed and agreed on the final semantic themes. Quantitative analysis was used as a comparison to qualitative analysis, measuring changes in levels of depression and anxiety which would be difficult to measure using only qualitative analysis [47]. The pre and post questionnaire scores were considered. A *t* test was used to compare the mean difference between the two groups’ scores.

For the quantitative evaluation, two standardised self-rating scales, the PHQ-9 [48] measuring low mood and depression, and the GAD-7 [49] measuring anxiety, were completed. The PHQ-9 is a 9-item, self-report with a Cronbach’s Alpha of 0.89 and has a maximum score of 27, with severity of depression measured as 0–4 none, 5–9 mild, 10–14 moderate, 15–19 moderately severe, 20–27 severe [48].

The GAD-7 is a 7-item, self-report with a Cronbach Alpha of 0.83 and scores ranging from 0 to 21: severity of anxiety 0–5 none, 6–10 mild, 11–15 moderate, 16–21 severe [50].

Measures were administered by hard copy pre and post programme. Reminders were sent to students who failed to return their questionnaires after 12 weeks.

## 3. Results

### 3.1. Qualitative Findings

Student focus groups were completed for Groups 1–3. The focus groups were recorded, transcribed and analysed, identifying three main themes: ‘Creating a safe community’, ‘Making progress’ and ‘Pathways to success’. No differences were identified between male and female participants. The qualitative survey results were used to gather the views of the student sports activators and staff who had referred students to the group, and were summarised.

#### 3.1.1. Theme 1 Creating a Safe Community

Respondents were asked about their lived experience of the group and how it affected their wellbeing. Four subthemes emerged: ‘a safe place and community’ (N = 28), ‘a shared experience’ (N = 23), and ‘commitment to group and self’ (N = 20).

Responders noted the group was a safe place with a relaxed enjoyable atmosphere: “It was the contrast of this is a really safe space… When I was walking home it often hit me the reality of, oh my god, I’ve got to go back to those people” (Group 2), “*it’s encouraging knowing…there are other people like you and it’s not some kind of disease*.” (Group 1), “*it’s a very comfortable relaxed kind of thing.*” (Group 3). The safe place was fostered by Rogerian principles of kindness used by the counsellor and staff and a non-judgemental atmosphere (Rogers, 1993): “…*I didn’t have to worry about social expectations. Like there was none of that; it was kind of just, you are who you are*” (Group 1).

Community and belonging were very prominent in the coding (N = 28). The MINDFIT group provided a break from their course, their established friendship groups, housemates and allowed permission to take a break from studying…“*I’d often bring something that was really stressing me that day and I could just speak to someone who’s not in your course, not a family member, obviously we’re not strangers to each other, but I think when it’s someone new and you know they can understand, and someone can reassure you as well*” (Group 1)…“*I live with people who are on my course, so it feels like for months and months and months the only thing we’ve talked about is work and dissertation…its nice (MINDFIT) because you’re talking about something different…*” (Group 1).

Specifically, the meeting with the counsellor before being introduced to the group was important “*meeting you beforehand…like broke down that barrier…I knew who you were before we met (the group) …it’s fine…you’re here and you recognised me. I’m in the right place*” (Group 2).

A sense of community was noted “*we made this community…a place to always go to if you need,…it is ownership*” (Group 2). Shared experience noted the similarity within the group: “*… our mental health impacts us, I think that was just a sense of unity and it gave me a feeling of togetherness*” (Group 1) and “*the sort of unspoken sameness… knowing that everyone here has probably struggled with something*” (Group 3).

Commitment to group and the self were also reported: “*I’d made a commitment to the group, without vocalising that in any real way*” (Group 2) and “…*it felt important to stick with it*” (Group 2) and students found morale and motivational support from the group “*… I probably wouldn’t have run at all over the last weeks if it wasn’t for the group and the motivation and support that you get from all the others around you*” (Group 2).

#### 3.1.2. Theme 2 Making Progress

Making progress relates to students perceiving that they were developing positively and holistically from MINDFIT, with the subthemes which reported on ‘normalising emotions’ accepting help’ (N = 33), ‘academic progression’ (N = 23), and ‘feeling more attuned to their wellbeing’ (N = 21).

Normalising emotions and accepting help were reported as important to participants: “*…it’s kind of scary…. like I’ve got a problem, but then it’s almost you’re glad that you’ve done it, you’re accepting the help … so you should be proud*” (Group 1) and “*it’s progress towards getting it dealt with*” (Group 2). Participants noted the physical wellbeing and mood elevation post run: “*I had three hours of feeling good … I was grateful for the three hours that I did have when I felt better*” (Group 2), “*I always felt like I slept better*” (Group 2) and “*at the end of a run, like, runners high…the endorphins going through your system, it actively makes me feel a lot calmer and a lot better*” (Group 3).

Responders also commented on their academic achievement and progression*…“I actually failed my first attempt of second year…I wasn’t doing any exercise, I wasn’t getting out and meeting new people…once I started running, like my dissertation…I was doing an hour every night after the running, and I managed to get 78 per cent…I don’t think I would have been as alert and on it if I hadn’t had something else to focus on*” (Group 2).

There was also an improved focus and determination to get on with studies: “*…you think, well, I’ve done a run…right, I’ve done that, so I can get on with my work*” (Group 3) and “*I’ve got better grades this year than I did last year. Because I feel like I’ve had that time to sort of relax a bit*” (Group 3).

#### 3.1.3. Theme 3 Pathways to Success

‘Pathways to success’ captures how students perceived MINDFIT helped them be more successful. Sub-themes included ‘a having a routine and structure’ (N = 34), “being productive and motivated’ (N = 32), ‘counsellor led programme’ (N = 21), ‘developing self-belief’ (N = 16) and ‘effective communication throughout’ (N = 25). These were a key contributing factor to a perceived improved university experience and sense of belonging.

Students noted that their routine and structure was enhanced by the MINDFIT group: “*having a set time was very important … just knowing that every event will be quite similar … like I can’t go up in different places all the time … it created a better relationship*” (Group 1). This group belonging was important in encouraging and shared experience. Although students might seem busy and appear to have contact with lot of people in a week, the quality of that contact did not necessarily give them understanding of their needs and some perceived they were lonely: “*Just having positive interactions on that day sort of changes your view of the week as a whole…loneliness is a bit of a killer*” (Group 3).

As well as these boundaries and structure, they noted an improvement in their productivity and personal motivation: “*I just ran for me … and it was like… I’ve got the motivation; I can look after myself and things like that*” (Group 2). There was a recognition that running in green space enhanced their experience “…*I think this programme and being outside and being with people in the fresh air, exercising…it’s just so much healthier*” (Group 1) and “…*you’d see all the daffodils…you’re running and take in some of the nature around as well*.” (Group 2).

Students also noted a change in their self-belief… “*It’s huge…when I first started coming here, I was seriously thinking of not finishing (PhD)… if I’ve got to choose between this and my sanity, I’m going to choose my sanity… and now I’m thinking I don’t have to choose I can finish this thing*” (Group 1).

Expectations and sense of safety were noted to be influenced by the inclusion of a counsellor: “*If it wasn’t for you as a counsellor and if it was just a running group, I would assume everyone is, like super fit*” (Group 1). Students stated that the counsellor was “*sensitive and emotionally proficient…fully involved and cares enough*” (Group 3).

Being part of MINDFIT appeared to enhance the university experience and belonging and increased self-belief. One student who was encouraged to remain in the group, whilst on a break in study, noted the group had “*been the most amazing thing I have been a part of…has kept me connected to my student identity…helped me to come back to studying…it gave me purpose and belonging*” (Group 1). For some students, the psychoeducation and talks by the founder of Sheffield Parkrun, focusing on resilience and perseverance, also contributed to an improved sense of self-belief: “*that’s probably the most beneficial thing I got out of the talk—as in the sort of not giving up …if you want to make something great, you’re going to have to face barriers*” (Group 1).

Effective communication arose as a particularly important factor for maintaining the connection between sessions and increasing the experience of a supportive community. Pre- and post-run texts were sent every week to the students and were written in a person-centred friendly tone, providing information and encouragement. Texts were informative, giving confidence to students who doubted themselves to turn up: “*oh like is it on…It’s like, ooh, just a good—You know?*” (Group 3), but they were also motivating, resulting in reduced anxiety and consolidated goals and achievements week by week. This collective acknowledgement appears to have fostered inclusivity and maintained a thread between sessions “*like a mini boost in the week*” (Group 1), “*going back through them all and just thinking oh, look how far you’ve come*” (Group 1) and “*Well done guys! That’s a nice little pat on the back*” (Group 3).

#### 3.1.4. Staff Voice

Wellbeing and academic staff commented on the perceived positive effect of the group on student mental health, including managing their problems, practicing new skills in a safe environment, “*pushing their boundaries in an understanding environment where they know support will be available*” and, in some cases, adding value to one-to-one therapeutic sessions by “*processing the new learning*” and “*maintaining work achieved in one-to-one therapeutic sessions”.* Time in Green space, daylight and a routine were also stated as beneficial to students.

#### 3.1.5. Student Sports Activators

The three Student Sports Activators were integral to the organisation of the group. Two had completed the programme as participants of the first group and one volunteered through the university sports activator programme. They reflected on the “*positive difference it made to students*” lives. They also acknowledged how the activator role had significantly helped their own mental health: “*This activator role has significantly improved my mental health whilst living and studying at university. This is because it has given me the chance to socialise and feel part of a community*”.

### 3.2. Quantitative Findings

**Figure 1:**
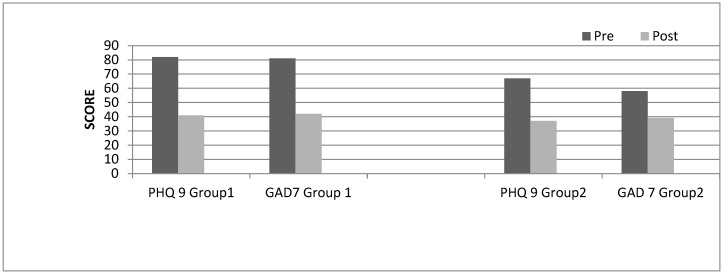
PHQ-9 and GAD-7 pre- and post MINDFIT measures. **Note** total scores were used.

The quantitative measures PHQ-9 and GAD-7 were collected pre and post intervention from Groups 1 and 2. Pre group data was collected for Groups 3 and 4 but due to COVID-19, post group data was not collected. Of the six students completing the programme in Group 1, paired t-test analysis, using totalled scores, showed a decrease on both the PHQ-9 (mean Time 1 = 82; mean Time 2 = 42; *p* < 0.01) and the GAD-7 (mean Time 1 = 81; mean Time 2 = 42; *p* < 0.05), demonstrating increases in perceived wellbeing (see Figure 1). For Group 2, pre and post PHQ-9 and GAD-7 data were collected from five of the eight students who completed the programme. Individual student identifiers were not used for Group 2; therefore, t-test analysis was not undertaken (see Figure 1).

**Group 1** PHQ-9 pre scores were 82/162 (51%) reduced to 42/162 (26%) for six students. GAD-7 pre scores were 81/126 (65%) reduced to 42/126 (33%) for six students. **Group 2** PHQ-9 pre scores 67/135 (50%) were reduced to 37/135 (27%) for five students. GAD-7 pre scores 58/105 (55%) were reduced to 39/105 (37%) for five students.

## 4. Discussion

Findings show how it is possible and beneficial to combine a therapeutic approach with a physical activity such as running.

Qualitatively, a multi-layered therapeutic effect was identified from the Themes 1, 2 and 3, whereby mastery of a goal contributed to an increase in self-belief. Feelings of community, belonging and safety were perceived to reduce individual anxiety and social anxiety, reducing isolation and improving perceived academic achievement and progression. These findings parallel Grant et al.’s [51] organised walking group outcomes, as MINDFIT led to negative beliefs being changed and new behaviours being developed. For example, the experience of parkrun developed an understanding of perseverance in the face of adversity and of sticking to personal goals rather than comparing with others.

Themes 1 and 2 show that students found solutions to problems within the group without consequence to their course, friendship groups or their course study groups. The group provided a safe place to normalize difficult emotions and a sanctuary whereby the group was contained and provided respite from everyday living [52,53]. Doughty [53] describes such temporary social outlets as having restorative value. Weekly access to therapeutic landscapes—greenspace [35]—may have also acted as a co-facilitator to restoration, providing an enabling environment, reducing stress and increasing wellbeing [35,54].

The combined effect of the natural environment and physical movement releases a greater capacity and access to personal social resources [52] and increases the fluidity of interactions [53]. Interactions between participants and facilitators of MINDFIT were dynamic, with interruptions, pauses, breaks in conversation, a “give and take” and “swinging along with places” (Wunderlich, [55] p. 132), allowing for fluidity which was facilitated through the running element of MINDFIT. This allowed students to interact with little eye contact (a key difficulty for students with anxiety and social anxiety) and thus removing pressure and providing a healing space.

MI was used as a coaching tool to improve motivation which affected participation and built confidence. Students weighed their perceptions of the perceived pain of running and the anxiety experienced as compared with the benefits they may experience [56,57]. The aim was to build trust and confidence in the philosophy of the group at triage and build on this week by week. Many of the students had never run before so had no experience of what it would feel like and of the benefits. The findings suggest that people move through contexts, thinking, feeling and adapting as per Tramonti [58]. This was enhanced by the presence of a counsellor who set the tone and non-competitive spirit to create the best conditions and context for change to occur, and build self-efficacy [57], helping develop Social Capital [59] where trust and kindness was displayed within and beyond sessions [51].

Theme 1 demonstrates the participants’ support for each other; for example, students were aware of other members’ safety by walking home together on the darker winter evenings. They encouraged each other, being genuinely pleased when people achieved another milestone in their running. This encouragement extended to a student led Facebook group, supporting each other during exams and thus building their community and social resources [59,60]. Trust developed and members developed their interpersonal skills, allowing some of their social anxiety to dissipate, and, through this, members developed new lived experiences and healing narratives. Personalised programme communications enhanced this bonding with students reporting the value added by the communication style, tone and content (theme 3).

Quantitatively, there was a promising effect on wellness scores on both the PHQ-9 and GAD-7 (see Figure 1). Reductions in anxiety and depression symptomology were seen over the groups evaluated. This echoes previous evidence that shows that PA combined with a psychological intervention can help students create a buffer against the stress factors of university study [61], along with how students perceive themselves in relation to the academic demands of their courses [62].

Overall, findings suggest that being part of MINDFIT positively affected students perceived academic achievement and progression. The regularity and structure of the group provided a contrast and a break from studies, but this also energised and increased motivation for studies. The self-belief gained through mastering a weekly goal transferred to academic progression, with displays of enhanced determination that may affect retention [63]. Theme 3 shows the combination of structure, and an informal friendly approach works well, giving students a positive focus and a stable point of communication each week where they know what is happening, which they feel they lack in their academic schedules and day-to-day life, especially as university and part time job timetables change frequently. Most participants strongly expressed they would never join a mainstream group or society as their general and social anxiety would be a barrier to this. These views align with the Theory of Planned Behaviour [63], in that the barrier is the students’ perception of their own competence, both their fitness and ability to form relationships.

Even though recruited over three stages, the small sample overall of people negates any chance of generalisability of the data from statistical results, although the qualitative findings do give some credibility and transferability for other UK and overseas universities. Data returns were affected by some students’ reliability to complete all their forms. The later groups were directly affected by COVID-19 lockdown. Likewise, Group 4, which had completed three sessions, was cancelled because of COVID-19 restrictions.

More female students took part in the programme than males which compares with the gender split for students accessing Student Wellbeing. Wellbeing is 1:2.6 male to female and MINDFIT 1:3.8 male to female. MINDFIT was also not inclusive of physically disabled students which is something to consider in future iterations.

This article’s evidence shows positive results but with limited numbers. However, we are in conversation with other UK universities about scaling up the programme, with the aim of a larger dataset to provide new insights and, of course, transformative help to the student participant.

## 5. Conclusions

MINDFIT provided an innovative therapeutic approach that demonstrates significant benefits. The triage process was an important first step towards building commitment and creating safety. An informal and structured communication style throughout the programme was also identified as helping to maintain this, as was the presence of an emotionally sensitive practitioner. Having a counsellor and mental health professionals involved provided a sense of emotional safety. Overall MINDFIT’s positive results are encouraging and a reason to continue this work and more in-depth evaluation with high-class research.

MINDFIT showed sustained participation with continued engagement from students after completion of their first programme, including volunteering to give back to the programme. Students built confidence in their social skills, reduced social and general anxiety on a weekly basis, with an overall outcome of improved perceived academic achievement and progression. We would recommend that other higher education establishments consider this programme as part of their wellbeing offer.

## Figures and Tables

**Table 1:** Sessional Psychoeducation.

Week	Content
1	Meet the group. Reiterate the MINDFIT philosophy. Share what you want to get from MINDFIT. Walking session, give shirts. Create belonging
2	Meditation and group chat about wellbeing. Feedback on the student experience
3	Physio input—strength and conditioning and stretching
4	Meditation and group chat—general about wellbeing and being part of a group creating community/feedback about their experience of the group
5	Cognitive Behavioural Therapy five system
6	Meditation group chat/feedback—spontaneous wellbeing topics
7	Mindfully strong speaker and activities
8	Hallam Parkrun founder talked about the parkrun philosophy, community and resilience needed when he set it up
9	Arrangement/preparation for parkrun and dealing with pre-event nerves
10	Arrangement/preparation for parkrun and dealing with pre-event nerves

**Table 2:** Student Groups.

Group	Referred	Triaged	Started	Completed	Non Completed
Group 1	16	9	9	6	3
Group 2	25	11	9	9	0
Group 3	21	13	10	9	1
Group 4	27	17	15	Canc	N/A
Totals	89	50	43	24/28 *	4

* Due to COVID-19, 24 out of 28 Completed in total, Groups 1 to 3. Group 1 all females. Group 2 mixed gender. Age of students in all groups, over 18.

## Data Availability

The datasets analysed during the current study are not publicly available yet because this data will be used to prepare at least one additional publication after which data will be publicly available. Currently, data are available from the corresponding author on reasonable request.
